# Evaluating Extended Curettage and Adjuvant Therapy Against Wide Resection and Reconstruction in the Management of Distal Radius Giant Cell Tumors: A Systematic Review and Meta-analysis

**DOI:** 10.1177/15589447241245736

**Published:** 2024-04-23

**Authors:** Ishith Seth, Gabriella Bulloch, Bryan Lim, Yi Xie, Nimish Seth, Warren M. Rozen, Sally Kiu-Huen Ng

**Affiliations:** 1 Department of Plastic Surgery, Peninsula Health, Melbourne, VIC, Australia; 2 Central Clinical School, Monash University, Melbourne, VIC, Australia; 3 Faculty of Medicine, Dentistry and Health Sciences, The University of Melbourne, VIC, Australia; 4 Department of Orthopaedic Surgery, Peninsula Health, Melbourne, VIC, Australia; 5 Department of Plastic Surgery, The Austin Health, Melbourne, VIC, Australia

**Keywords:** giant cell tumor, GCT, radius, curettage, wide resection

## Abstract

**Background:**

The management of distal radius giant cell tumors (GCTs) remains challenging, and the optimal approach is still a matter of debate. This systematic review and meta-analysis aimed to compare the outcomes of extended curettage and wide resection, the mainstays of treatment.

**Methods:**

Medline (via PubMed), Cochrane Library, Web of Science, Google Scholar, ClinicalTrials.gov, and Embase databases were searched for comparative studies that assessed extended curettage with adjuvant therapy and wide resection with reconstruction in patients with GCTs of the distal radius up to April 2023. Data were collected and analyzed on rates of local recurrence, metastasis, overall complications, and functional outcomes. The Newcastle-Ottawa scale was used to appraise the risk of bias within each study.

**Results:**

Fifteen studies (n = 373 patients) were included and analyzed. Patients who underwent curettage were more likely to develop recurrence (risk ratio [RR] = 3.02 [95% confidence interval; CI, 1.87-4.89], *P* < .01), showed fewer complications (RR = 0.32 [95% CI, 0.21-0.49], *P* < .01), and showed greater improvement in Visual Analog Scale and lower Disabilities of the Arm, Shoulder, and Hand scores (*P* < .00001) than those who underwent wide resection. No significant difference was found regarding metastasis (RR = 1.03 [95% CI, 0.38-2.78], *P* = .95).

**Conclusions:**

Regarding the surgical approach to GCT of the distal radius, curettage with adjuvant therapy was associated with a higher likelihood of recurrence compared with wide resection with reconstruction. Nevertheless, the curettage approach resulted in significantly lower rates of operative complications, decreased pain scores, and better functional outcomes in comparison to the resection group.

## Introduction

Giant cell tumors (GCTs) of the bone are neoplasms that exhibit a significant propensity for local invasiveness and a marked incidence of local relapse.^1^ Giant cell tumors account for around 5% of primary bone tumors and 20% of benign bone tumors. Most cases are diagnosed between the ages of 20 and 40 years, with a slightly higher incidence in women.^1^ They are commonly located in the metaphyseal-epiphyseal region of long bones, with the distal femur and proximal tibia being the most frequently affected sites, accounting for 50% to 75% of cases.^2,3^ Approximately, 10% to 12% of GCT cases occur in the distal radius, where there is a higher risk of local recurrence.^4,5^ Pulmonary metastases from GCTs represent approximately 1% to 9%, and the risk of mortality in cases of pulmonary metastases remains low.^6-8^

The surgical management strategies for GCTs of the distal radius include extended curettage and wide resection with reconstruction.^9^ The ideal surgical approach for GCTs remains a subject of controversy. In cases where the tumor is confined to the bone, favorable outcomes have been documented following extended curettage and adjunctive therapy.^10^ Should the tumors present with soft-tissue masses, cortical destruction, and joint invasion, the preferred treatment approach involves wide resection of the tumor, followed by wrist joint reconstruction using either an osteoarticular graft or arthrodesis.^11^

Previous studies have indicated that wide excision with resection results in lower rates of recurrence.^12,13^ However, resections necessitate sacrificing the radiocarpal and distal radioulnar joints, which presents a significant challenge for reconstruction due to the complex functional requirements of the wrist joint. This approach also carries high rates of complications and poor functional outcomes.^14,15^ In contrast, extended curettage has been found to enable the preservation of the joint, maintenance of wrist function, and enhanced patient-reported outcomes in contrast to wide resection.^12,13^

A prior meta-analysis by Liu et al^16^ showed 6 assessed studies, comprising a total of 59 and 80 cases for wide resections and curettage, respectively. The findings indicated that, despite a greater relative risk of local recurrence among patients treated with curettage, this group achieved superior functional outcomes.^13,17^ A further meta-analysis conducted by Pazionis et al, comprising 6 studies, yielded similar results.^12^ Given the significant interest in this area of research, an updated meta-analysis is necessary to consider the substantial amount of new information that has emerged in managing this complex tumor.

The purpose of this meta-analysis was to compare the outcomes of extended curettage with adjuvant therapy and wide resection with reconstruction. The study aimed to determine the recurrence and metastasis rates for each surgical option. In addition, the study explored the differences in complications, postoperative function, and pain scores between the two surgical approaches.

## Methods

The current study adhered to both the Cochrane Handbook of Systematic Reviews of Interventions and the Preferred Reporting Items for Systematic Reviews and Meta-Analyses (PRISMA) statement guidelines throughout all stages (Supplemental Table 1).^18^ The study was registered on PROSPERO, the International Prospective Systematic Review (CRD420212422614).

### Literature Search Strategy

Medline (via PubMed), Cochrane Library, Web of Science, Google Scholar, ClinicalTrials.gov, and Embase from January 1901 to April 2023 were used. We employed the possible combinations of the keywords: “giant cell tumor,” “distal radius,” “curettage,” and “resection.” In addition, the reference lists of relevant articles were manually reviewed. Supplemental Table 2 includes a comprehensive overview of the search strategies employed.

### Eligibility Criteria

In this systematic review and meta-analysis, studies were included if they met the following criteria: (1) They were comparative studies that compared wide resection with reconstruction and extended curettage with adjuvant therapy for GCTs specifically in the distal radius; (2) they were conducted on human participants; and (3) they were written in the English language. There were no restrictions on the minimum number of cases or duration of follow-up. Extended curettage with adjuvant therapy refers to a surgical procedure where the tumor tissue is removed using a curette along with the surrounding healthy tissue to ensure complete excision. Adjuvant therapy, which can include various substances like phenol, polymethyl methacrylate, or liquid nitrogen, is then applied to the site to destroy any remaining tumor cells and reduce the risk of recurrence.

Studies were excluded if they were noncomparative, included other joints, or did not report the outcomes of interest. Furthermore, we excluded animal studies, review articles, case reports, conference abstracts, non–English-language studies, and duplicate references from the analysis.

### Study Selection

Titles and abstracts of studies identified during the search were imported into Endnote X20 for preliminary screening. Full texts of potentially relevant articles were further screened using the eligibility criteria. These were done by two independent reviewers (IS and GB), and any disparity in either selecting eligible studies or assessing findings between the two reviewers was resolved through consultation with the rest of the authors.

### Data Extraction

Two independent authors (IS and GB) gathered the necessary data into a designated data extraction form. The acquired information encompassed the following parameters: study design, number of patients in each group, mean age, male-to-female ratio, follow-up, Campanacci grade, intraoperative adjuvant therapy, defect following curettage, and reconstruction following resection.

The incidence of recurrence, metastases, and mortality were recorded, as well as patient-reported outcomes such as pain scores (using Visual Analogue Scale [VAS] score from 0 to 10) and functional outcomes based on the Disabilities of Arm, Shoulder, and Hand (DASH) score^6^ and Musculoskeletal Tumor Society (MSTS) score.^19^ The overall complication rates were also extracted and documented.

### Risk of Bias Assessment

The Newcastle-Ottawa scale^7^ was used to evaluate the risk of bias in the observational studies included in our analysis. This instrument assesses each study reporting 3 domains. First, the selection of study participants must be representative of the population and the diagnosis of GCTs should be ascertained using predefined criteria. Second, groups should be comparable regarding demographic characteristics and relevant confounders. Finally, prespecified outcomes must be ascertained with sufficient follow-up duration.

### Data Synthesis

For dichotomous data, risk ratios (RRs) with a 95% confidence interval (CI) were used. For continuous data, mean differences (MDs) with a 95% CI were employed. Data synthesis was performed using R software (meta-package, Version 6.2-1, https://www.r-project.org/, Lucent Technologies, USA). Visual inspection of the forest plots and measurement of the *Q* statistic and *I*^2^ statistic were used to assess heterogeneity. Significant statistical heterogeneity was indicated by *Q* statistic (*P* < .1 or *I*^2^ >50%). In the event of significant heterogeneity, a random effect model was employed; otherwise, a common-effect model was used. Sensitivity analyses were conducted by removing one study at a time to identify potential sources of heterogeneity. Subgroup analyses were also planned. A funnel plot was used to assess publication bias for outcomes with more than 10 studies.

### Quality of Evidence

The assessment of the strength of recommendations and evidence in the current study was independently conducted by two reviewers using the Grading of the Recommendations Assessment, Development, and Evaluation (GRADE) Handbook. This framework evaluates the risk of bias, inconsistency, indirectness, imprecision, and publication bias, with quality levels categorized as “high,” “moderate,” “low,” or “very low.”^8^

## Results

**Figure 1.**
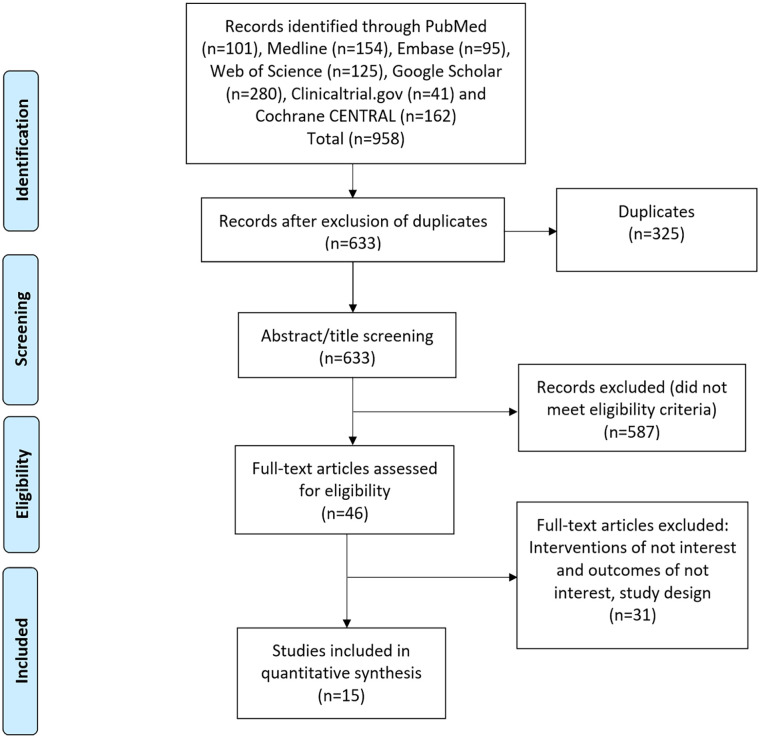
Preferred Reporting Items for Systematic Reviews and Meta-Analyses checklist of study selection.

We identified 633 distinct records through a comprehensive literature search. After screening the titles and abstracts, we retrieved and examined 46 articles to determine their eligibility, ultimately including 15 studies in our final analysis. A PRISMA flow diagram was created to illustrate the selection process (Figure 1).

### Characteristics of Included Studies

Table 1 depicts the characteristics of the 15 retrospective comparative cohort studies^9,12-17,20-28^ that met our inclusion criteria, published between 1993 and 2022. The total number of procedures analyzed was 471, with 240 of those using extended curettage with adjuvant therapy and 231 undergoing wide resection with reconstruction. The average age of participants was 33.49 (SD ±2.3) years, and the average follow-up period was 7.1 (SD ±3.7) years. Further features of the included studies, such as the use of intraoperative adjuvant therapy, the method of defect filling following curettage, and the type of reconstruction after resection, are reported in Supplemental Table 3. The risk of bias appraisal indicated a moderate to high risk of bias and low to moderate overall quality.

**Table 1.** Summary of Included Studies.

Study	No. of patients	Age, y	Sex ratio M:F	Follow-up, y	Campanacci grade	Intraoperative adjuvants	Defect filled following curettage	Reconstruct following resection
Abuhejleh et al^15^	57	35.4	25:32:00	7.2	II = 13, III = 40a	Burring followed by inconsistent use of adjuvant and followed with jet wash	No. 23: bone cement No. 10: bone graft No. 1: empty	No. 7: vascularized fibular autografts No. 16: nonvascularized autograft
Atalay et al^13^	20	28.6	6:14		I = 1, II = 13, III = 2	Burring, ethanol	Autologous fibular graft, allogeneic bone graft segment, iliac graft, or combinations	Allograft fibula + iliac autograft
Chanchairujira et al^22^	10	31	32:42	3.2	NR	Cement or bone graft	NR	NR
Cheng et al^26^	12	35	4:08	6.8	III = 12	High speed burr and phenol	No. 6: autogenous cancellous bone graft, which was harvested from the iliac crest	No. 4: osteoarticular allograft No. 2: fibular autograft
Harness and Mankin^25^	46	31	NR	14	I = 3, II = 33, III = 10	Burring or phenolization and insertion of PMMA	No. 5: autograft No. 26: PMMA cement	NR
Jiao et al^12^	32	NR	13:19	2.5	II = 11, III = 10	Microwave ablation	Bone cement filling	Nonvascularized autologous fibula reconstruction
Kang et al^23^	15	38	10:05	5	III = 15	High-speed burring of the endosteal cavity, followed by irrigation, drying, and electrocautery coagulation ± liquid nitrogen	Antibiotic-laden PMMA cement	No. 5: patients with vascularized or neovascularized intercalary fibula autogenous graft arthrodesis No. 1: total wrist arthroplasty/allograft composite
Mozaffarian et al^17^	13	33.7	6:07	6	III = 13	High-speed burring	Irrigation with saline and filling with bone cement	Proximal fibular autograft
Panchwagh et al^24^	24	36	13:11	3.1	I = 1, II = 9, III = 14	Phenol	No. 5: bone graft No. 4: cement No. 2: no form of reconstruction	Proximal fibular graft wrist arthrodesis
Sheth et al^27^	26	34	12:14	9	I = 2, II = 8, III = 16	Liquid nitrogen	No. 9: bone graft No. 7: PMMA cement No. 2: no form of reconstruction	The selection of bone for arthrodesis varied from tibial cortex, tricortical iliac crest, ulna, or fibula
van der Heijden et al^9^	77	NR	41:35	8.8	NR	Phenol, PMMA, liquid nitrogen, soft-tissue extension	NR	No. 17: osteoarticular allograft No. 9: primary arthrodesis No. 2 fibula-pro-radius
Vander Griend and Funderburk^28^	22	31.8	5:17	5:03	NR	High-speed burn, combined more recently with pulsating lavage and electrocautery + PMMA	Packing with cement	No. 6: nonvascularized autogenous bone graft from the fibula No. 4: the adjacent ulna No. 1: the iliac crest
Wysocki et al^21^	39	34	22:17	11.3	II = 15, III = 24	No. 20: burr exteriorization No. 10: phenol No. 12: electrocautery No. 4: argon beam No. 19: PMMA No. 1: cancellous allograft	NR	No. 6: nonvascularized fibular autograft No. 3: distal radius allograft No. 3: ulnar transposition No. 3: fibular allograft
Zhang et al^20^	20	34.8	NR	2.1	NR	95% ethanol was used to inactivate the tumor bed	Allogeneic bone graft/bone cement augmentation	Autologous fibular graft/allogeneic bone graft
Zou et al^14^	58	33.2	35:23	7.9	NR	High-speed burring, iodine tincture, electrocautery	No. 9: PMMA cement No. 8: cancellous allograft No. 4: autograft	No. 26: fibular autograft No. 6: distal allograft No. 5: PMMA

*Note.* PMMA = polymethyl methacrylate; NR = not reported.

### Outcomes

#### Recurrence

**Figure 2.**
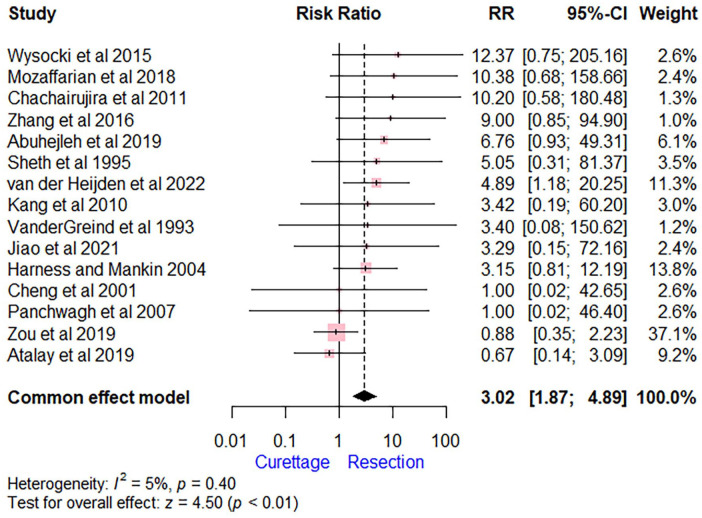
Forest plot comparing extended curettage with adjuvant therapy versus wide excision with reconstruction regarding recurrence. *Note*. RR = risk ratio; CI = confidence interval.

Sixty-six of 232 patients (28.4%) in the curettage group and only 17 of 220 patients (7.7%) in the wide resection group experienced recurrence. The pooled estimate showed a higher recurrence rate in the curettage group compared with the resection group (RR = 3.02 [95% CI, 1.87-4.89], *P* < .01). The *I*^2^ score of 5% indicates low heterogeneity among the studies (Figure 2).

#### Metastasis

**Figure 3.**
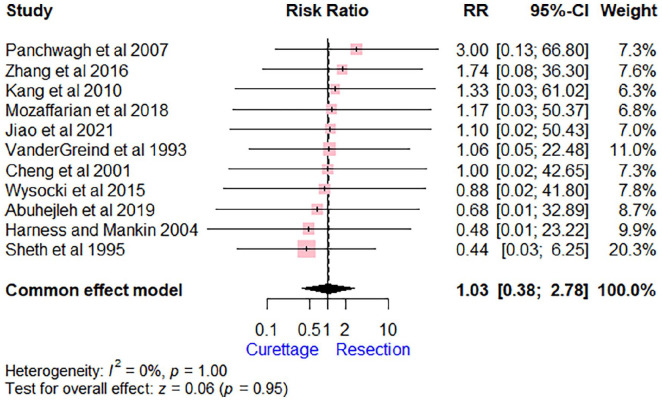
Forest plot comparing extended curettage with adjuvant therapy versus wide excision with reconstruction regarding metastasis. *Note*. RR = risk ratio; CI = confidence interval.

The incidence of distant metastases was 1.3% (2/152) of those in the curettage group and 2% (3/149) in the resection group. The pooled estimate showed no significant difference for developing metastasis following either type of surgery (RR = 1.03 [95% CI, 0.38-2.78], *P* = .95). The *I*^2^ score of 0% suggests low heterogeneity among the studies. Among the 5 patients who developed distant metastases, 4 had pulmonary metastasis, whereas 1 had a bony metastasis to the ipsilateral clavicle (Figure 3).

#### Complications

**Figure 4.**
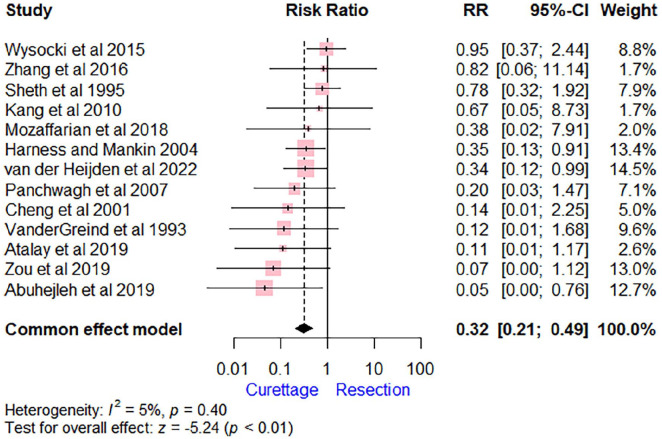
Forest plot comparing extended curettage with adjuvant therapy versus wide excision with reconstruction regarding complications. *Note*. RR = risk ratio; CI = confidence interval.

The surgical complications, including nonunion, malunion, fracture, and infection, were reported in 11.4% (25/220) in the curettage group and 36.7% (73/199) in the resection group. The pooled estimate showed a lower complication rate in the curettage group compared with the resection group (RR = 0.32 [95% CI, 0.21-0.49], *P* < .01). The *I*^2^ score of 5% suggests low heterogeneity among the studies (Figure 4). The most common complication in the resection group was nonunion (14/37, 39%), followed by postoperative fracture (9/37, 24%). In the curettage group, the most common complication was the collapse of the radiocarpal joint (10/25, 40%).

#### Functional outcomes

**Figure 5.**
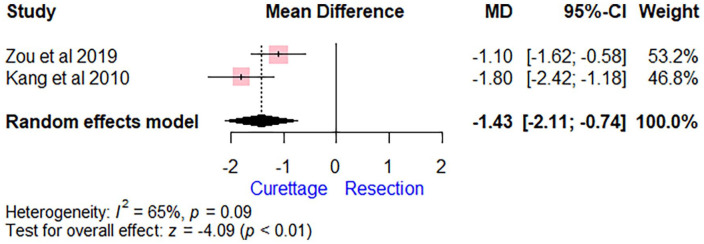
Forest plot comparing extended curettage with adjuvant therapy versus wide excision with reconstruction regarding Visual Analogue Scale pain scores. *Note*. MD = mean difference; CI = confidence interval.

**Figure 6.**
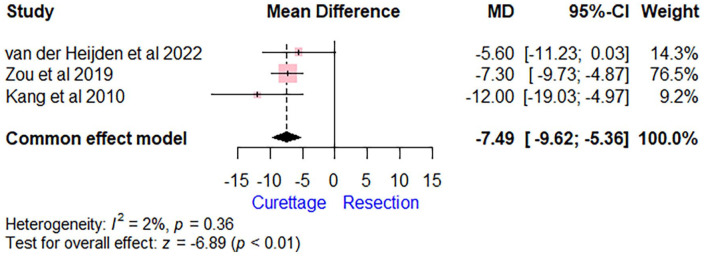
Forest plot comparing extended curettage with adjuvant therapy versus wide excision with reconstruction regarding Disabilities of Arm, Shoulder, and Hand score. *Note*. MD = mean difference; CI = confidence interval.

The analysis showed that extended curettage was associated with a significant VAS pain reduction (MD = −1.43 [95% CI, − 2.11 to −0.74], *P* < .01) compared with wide resection (Figure 5). The DASH score was significantly lower for patients who underwent extended curettage compared with those who had wide resection (MD = −7.49 [95% CI, −9.62 to −5.36], *P* < .01; Figure 6). Heterogeneity was observed in the analysis of VAS score (*I*^2^ score of 65%), whereas no heterogeneity was found regarding the DASH score (*I*^2^ score of 2%). Regarding the range of motion, the pooled estimate showed no significant difference between the compared groups for flexion/extension (MD = 4.59 [95% CI, −31.96 to 41.14]) and ulnar deviation/radial deviation (MD = −3.67 [95% CI, −30.62 to 23.28]). One study (Wysocki et al)^21^ reported a lower range of motion (pronation/supination) in the curettage group compared with the resection group (MD = −29.70 [95% CI, −44.15 to 15.25]; Supplemental Figure 1). The curettage group was associated with a higher grip strength (MD = 18.08 [95% CI, 13.78-22.37], *P* < .01; Supplemental Figure 2). The MSTS scores were reported inconsistently, and thus, pooled data analysis was not possible for the included studies.

#### Publication bias

To evaluate potential publication bias, a funnel plot was constructed for outcomes that had more than 10 studies. The results indicated no substantial evidence of publication bias regarding recurrence, metastasis, and complications as depicted in the Supplemental Material.

#### Quality of evidence

According to the evaluation approach by GRADE, the study results were related to a level of evidence ranging from moderate to high quality. None of the outcomes showed evidence of being associated with low or very low levels of quality. The specifics of the quality of evidence according to GRADE are elaborated in Supplemental Tables 4 and 5.

## Discussion

Giant cell tumors are a common musculoskeletal neoplasm in current orthopedic practice. Despite their benign nature, these tumors can be locally aggressive with a small likelihood of pulmonary metastases.^29^ Lesions in the distal end of the radius have a higher risk of local recurrence than those in other sites, possibly due to the anatomic geometry of the distal radius, lack of muscle coverage, complexity of the distal radioulnar joint, and proximity to the neurovascular bundle, flexor, and extensor tendons of the hand.^21,30^ Treatment of GCTs of the distal radius is challenging, as surgeons need to balance oncological clearance with function preservation. Previous meta-analyses have attempted to address this issue and have shown that although curettage is a less invasive procedure that preserves wrist joint function, wide resection has a lower recurrence rate, particularly in Campanacci grade III tumors.^16,30, 31^ More new comparative studies have been published, and we believe that an updated review is necessary due to the new information available.

Our study found a significantly lower recurrence rate with wide resection with reconstruction (7.7%) compared with extended curettage with adjuvant therapy (28.4%). Similar findings were reported by previous studies.^16,30, 31^ However, the option of curettage should not be dismissed for patients with grade III lesions, especially those with no involvement of the wrist joint, less than 50% cortical destruction, or an extraosseous mass visible from multiple angles. In such cases, extended curettage may still be the first line of surgical management, as recommended by Cheng et al^26^ and Kang et al.^23^ However, Mozaffarian et al disagreed with this approach and suggested that curettage should be reserved for only grade I and II GCTs of the distal radius due to the high recurrence rates observed in their study (66.7%).^17^ We believe that curettage is suitable for grade I and II GCTs of the distal radius because these lesions tend to have a delayed clinical presentation and require a more aggressive line of management.^15^ In addition, functional compensation provided by the uninvolved ipsilateral upper limb can partly compensate for the morbidity associated with resection.^15^ The current accepted rates for recurrence following extended curettage and wide resection are 31% to 35% and 0% to 8%, respectively.^32^

The current meta-analysis showed that extended curettage was associated with a significant reduction in the VAS pain scale and DASH score compared with those who had a wide resection, with no significant difference regarding the range of motion. Furthermore, the curettage group was associated with a higher grip strength compared with the resection group. In a retrospective analysis of 15 grade III GCTs of the distal radius, it was found that extended excision resulted in significantly higher grip strength and improved VAS scores when compared with resection.^22^ Cheng et al reported similar results when comparing the outcomes of curettage with wide resection and osteoarticular allograft in 6 patients.^26^ Patients in the resection group had a mean follow-up of 6 years and retained 69% of their wrist range of motion and 70% of their baseline grip strength, whereas no measurable differences were observed in the curettage group. However, Sheth et al found that both techniques had equivalent functional outcomes.^27^ Mozaffarian et al conducted a prospective evaluation of 13 distal radius GCTs and found that patients who underwent wide resection retained acceptable ranges of motion in flexion/extension and pronation/supination, which were 83% and 92% of those in the curettage group, respectively.^17^ Similarly, Wysocki et al found that the excision and resection groups had comparable MSTS scores.^21^

Distal radius resection complications have varying rates, including nonunion/malunion of the graft-radius junction, graft fracture, carpal subluxation, and degenerative arthritis of the wrist joint. Our review found that overall complication rates were higher with wide resection with reconstruction than extended curettage with adjuvant therapy. Several graft alternatives have been suggested, including nonvascularized and vascularized autografts as well as structural allografts.^14,19,23^ For the reconstruction, there are various options, such as different arthrodesis techniques or joint-preserving reconstructions like the Sauve-Kapandji type reconstruction.^33^ Nevertheless, no graft or reconstruction technique has been demonstrated to be superior, and all involve their advantages and disadvantages.^34,35^ Individuals who require wide resection and reconstruction must be made aware of the significant risks of complications and the possibility of subsequent surgeries associated with this procedure.

Although the focus of this review was not on the impact of denosumab on the management of GCTs, it is worth noting that its increasing use warrants discussion.^36,37^ Divergent opinions have been published regarding the effectiveness and safety of denosumab in the context of curettage or resection. Initial studies proposed that administering denosumab prior to surgery resulted in downstaging and did not elevate the incidence of local recurrence.^38,39^ However, recent data with extended follow-up have suggested that neoadjuvant denosumab is associated with an increase in the rate of local recurrence among patients undergoing curettage, and its function in the treatment of GCTs requires clarification.^40^

This analysis has several strengths, including its comprehensive nature, which included 15 studies, and the absence of significant heterogeneity and publication bias. However, there are significant limitations that need to be addressed. To begin with, this study is limited in its ability to address selection bias among patients who undergo resection versus curettage, as resection may be the only viable option for unsalvageable joints. It is plausible that in the studies included in this analysis, patients with more advanced disease and bony destruction received resection. Second, all the studies included in this analysis were retrospective and exhibited a moderate to high risk of bias for most outcomes. Finally, due to the inconsistencies in reporting, this study was only able to compare pain scores and disability ratings in a small subset of the studies included. As a result, the findings of this study should be interpreted with care.

## Conclusion

The employment of extended curettage with adjuvant therapy could potentially lead to higher recurrence rates; however, it may also provide improved functional outcomes in comparison to wide resection with reconstruction. It is crucial to consider the tumor’s severity and the patient’s unique circumstances when determining the most suitable treatment approach. To verify the most effective surgical treatment method for GCTs, future research should involve rigorously designed prospective randomized controlled trials.

## Supplemental Material

sj-docx-1-han-10.1177_15589447241245736 – Supplemental material for Evaluating Extended Curettage and Adjuvant Therapy Against Wide Resection and Reconstruction in the Management of Distal Radius Giant Cell Tumors: A Systematic Review and Meta-analysis Supplemental material, sj-docx-1-han-10.1177_15589447241245736 for Evaluating Extended Curettage and Adjuvant Therapy Against Wide Resection and Reconstruction in the Management of Distal Radius Giant Cell Tumors: A Systematic Review and Meta-analysis by Ishith Seth, Gabriella Bulloch, Bryan Lim, Yi Xie, Nimish Seth, Warren M. Rozen and Sally Kiu-Huen Ng in HAND

sj-docx-2-han-10.1177_15589447241245736 – Supplemental material for Evaluating Extended Curettage and Adjuvant Therapy Against Wide Resection and Reconstruction in the Management of Distal Radius Giant Cell Tumors: A Systematic Review and Meta-analysis Supplemental material, sj-docx-2-han-10.1177_15589447241245736 for Evaluating Extended Curettage and Adjuvant Therapy Against Wide Resection and Reconstruction in the Management of Distal Radius Giant Cell Tumors: A Systematic Review and Meta-analysis by Ishith Seth, Gabriella Bulloch, Bryan Lim, Yi Xie, Nimish Seth, Warren M. Rozen and Sally Kiu-Huen Ng in HAND

sj-docx-5-han-10.1177_15589447241245736 – Supplemental material for Evaluating Extended Curettage and Adjuvant Therapy Against Wide Resection and Reconstruction in the Management of Distal Radius Giant Cell Tumors: A Systematic Review and Meta-analysis Supplemental material, sj-docx-5-han-10.1177_15589447241245736 for Evaluating Extended Curettage and Adjuvant Therapy Against Wide Resection and Reconstruction in the Management of Distal Radius Giant Cell Tumors: A Systematic Review and Meta-analysis by Ishith Seth, Gabriella Bulloch, Bryan Lim, Yi Xie, Nimish Seth, Warren M. Rozen and Sally Kiu-Huen Ng in HAND

sj-tif-3-han-10.1177_15589447241245736 – Supplemental material for Evaluating Extended Curettage and Adjuvant Therapy Against Wide Resection and Reconstruction in the Management of Distal Radius Giant Cell Tumors: A Systematic Review and Meta-analysis Supplemental material, sj-tif-3-han-10.1177_15589447241245736 for Evaluating Extended Curettage and Adjuvant Therapy Against Wide Resection and Reconstruction in the Management of Distal Radius Giant Cell Tumors: A Systematic Review and Meta-analysis by Ishith Seth, Gabriella Bulloch, Bryan Lim, Yi Xie, Nimish Seth, Warren M. Rozen and Sally Kiu-Huen Ng in HAND

sj-tiff-4-han-10.1177_15589447241245736 – Supplemental material for Evaluating Extended Curettage and Adjuvant Therapy Against Wide Resection and Reconstruction in the Management of Distal Radius Giant Cell Tumors: A Systematic Review and Meta-analysis Supplemental material, sj-tiff-4-han-10.1177_15589447241245736 for Evaluating Extended Curettage and Adjuvant Therapy Against Wide Resection and Reconstruction in the Management of Distal Radius Giant Cell Tumors: A Systematic Review and Meta-analysis by Ishith Seth, Gabriella Bulloch, Bryan Lim, Yi Xie, Nimish Seth, Warren M. Rozen and Sally Kiu-Huen Ng in HAND
