# Supplementary material for: Evaluating Extended Curettage and Adjuvant Therapy Against Wide Resection and Reconstruction in the Management of Distal Radius Giant Cell Tumors: A Systematic Review and Meta-analysis
Source: Hand (N Y). 2024 Apr 23;20(7):1012–21. doi: 10.1177/15589447241245736 (PMC11571547; doi:10.1177/15589447241245736)
Supplement: sj-docx-5-han-10.1177_15589447241245736 – Supplemental material for Evaluating Extended Curettage and Adjuvant Therapy Against Wide Resection and Reconstruction in the Management of Distal Radius Giant Cell Tumors: A Systematic Review and Meta-analysis [file sj-docx-5-han-10.1177_15589447241245736.docx]

Supplementary Figure Legends

Supplementary Figure 1. Comparative analysis of pronation and supination range of motion in Curettage versus Resection treatment groups

Supplementary Figure 2: Evaluation of grip strength variations between Curettage and Resection treatment groups
